# The knowledge of Indonesian pediatric residents on hyperbilirubinemia management

**DOI:** 10.1016/j.heliyon.2021.e06661

**Published:** 2021-04-06

**Authors:** Mahendra T.A. Sampurna, Rinawati Rohsiswatmo, Aris Primadi, Setya Wandita, Eko Sulistijono, Arend F. Bos, Pieter J.J. Sauer, Christian V. Hulzebos, Peter H. Dijk

**Affiliations:** a Neonatology Division, Department of Pediatrics, Airlangga University Teaching Hospital, Faculty of Medicine, Universitas Airlangga, Surabaya, Indonesia; b Neonatology Division, Department of Pediatrics, Cipto Mangunkusumo Hospital, Faculty of Medicine, Universitas Indonesia, Jakarta, Indonesia; c Department of Pediatrics, Hasan Sadikin Hospital, Faculty of Medicine, Universitas Padjajaran, Bandung, Indonesia; d Neonatology Division, Department of Child Health, Faculty of Medicine, Public Health and Nursing, Universitas Gadjah Mada, Yogyakarta, Indonesia; e Department of Pediatrics, Saiful Anwar Hospital, Faculty of Medicine, Universitas Brawijaya, Malang, Indonesia; f Department of Pediatrics, Beatrix Children's Hospital, University Medical Center Groningen, University of Groningen, Groningen, The Netherlands

**Keywords:** Hyperbilirubinemia, Newborn infants, Knowledge, Residents, Guidelines

## Abstract

Hyperbilirubinemia in the newborn occurs more frequently in Indonesia. Therefore, it is important that pediatric residents in Indonesia acquire adequate knowledge of hyperbilirubinemia management. This study aims to determine the pediatric residents' knowledge on hyperbilirubinemia management, whether they follow recommended guidelines, and whether differences exist between five large Indonesian teaching hospitals. We handed out a 25-question questionnaire on hyperbilirubinemia management to pediatric residents at five teaching hospitals. A total of 250 questionnaires were filled in completely, ranging from 14 to 113 respondents per hospital. Approximately 76% of the respondents used the Kramer score to recognize neonatal jaundice. Twenty-four percent correctly plotted the total serum bilirubin levels (TSB) on the phototherapy (PT) nomograms provided by the American Academy of Pediatrics (AAP) and the National Institute for Health and Care Excellence (NICE) for full-term and nearly full-term infants. Regarding preterm infants <35 weeks' gestational age, 66% of the respondents plotted TSB levels on the AAP nomogram, although this nomogram doesn't apply to this category of infants. Seventy percent of residents knew when to perform an exchange transfusion whereas 27% used a fixed bilirubin cut-off value of 20 mg/dL. Besides PT, 25% reported using additional pharmaceutical treatments, included albumin, phenobarbitone, ursodeoxycholic acid and immunoglobulins, while 47% of the respondents used sunlight therapy, as alternative treatment. The limited knowledge of the pediatric residents could be one factor for the higher incidence of severe hyperbilirubinemia and its sequelae. The limited knowledge of the residents raises doubts about the knowledge of the supervisors and the training of the residents since pediatric residents receive training from their supervisors.

## 1 Introduction

Today's pediatric residents are tomorrow's pediatricians. Thorough pathophysiological knowledge and proper clinical training are essential ingredients for good pediatric care in the future. This also holds true for fast developing countries such as Indonesia. Clinical practice is evolving and moving away from eminence-based towards evidence-based medicine. As a consequence, clinical management will be optimized by developing guidelines based on scientific evidence, and this includes evidence-based guidelines on the management of hyperbilirubinemia.

In comparison to high-income countries, severe neonatal hyperbilirubinemia occurs more frequently in Indonesia [1]. Therefore, it is of utmost importance that pediatric residents in Indonesia acquire adequate knowledge about the diagnostic process and optimal treatment of hyperbilirubinemia. Lack of knowledge may result in inadequate management and, as a consequence, unnecessarily high rates of severe hyperbilirubinemia and adverse neurological outcomes, which could have been prevented. Studies in Canada have shown that the awareness of and adherence to guidelines on hyperbilirubinemia management help to reduce the incidence of severe hyperbilirubinemia [2,3].

Awareness of the existence of national guidelines and using them properly is therefore important. Our aim was to determine the level of knowledge of pediatric residents on the management of neonatal hyperbilirubinemia, whether they follow the recommended guidelines, and whether there are differences between the residents of different teaching hospitals.

## 2 Material and methods

During a six-week period, from December 2016 to January 2017, questionnaires on hyperbilirubinemia management were handed out to pediatric residents who had passed at least the junior neonatology internship in the five largest academic teaching hospitals in Indonesia. In each hospital a Person in Charge (PIC) was assigned who received by mail the file, copy and had to print it and to hand it handed out to their residents in their hospital. Prior to handing out the questionnaires to participants, written consent was acquired from all participants in training, provided that they agreed to spend time answering the questionnaires. All of the included hospitals are Academic Teaching Hospitals that serve as the highest referral facility in each of the encompassed areas. According to the regulation of the Child Health Collegium at least 3 neonatology consultants work in each Academic Hospital. Each hospital has their own protocols or guidelines on patient management as accredited by the Indonesian hospital accreditation committe. In Indonesia, clinical internship is preceded by one year of lectures on classical theory followed by examinations. The academic teaching hospitals were located in Jakarta, Bandung, Malang, Yogyakarta, and Surabaya. The questionnaire consisted of 25 questions on hyperbilirubinemia management and the corresponding response options [Table 1]. These varied from a simple choice between “Yes” or “No“ to choosing an answer among several options. This study approved by the IRB Dr. Soetomo General Hospital number 390/Panke.KKE/V/2017.

**Table 1:** Questionnaire on the management of hyperbilirubinemia for Indonesian residents in pediatrics.

No	Question	Answer	Number	%
1	What is your definition of hyperbilirubinemia?	TSB >5 mg/dL at24 h of age, 10 mg/dL at 48 h of age	49	19.9
		TSB >10 mg/dL for full-term and TSB >5 mg/dL for preterm	63	25.6
		TSB > P_95_ according to hours of age (Bhutani nomogram)	78	31.7
		TSB P_40_–P_75_ according to hours of age (Bhutani nomogram)	15	6.1
		TSB > P_5_ according to hours of age (Bhutani nomogram)	20	8.1
		Clinical assessment with jaundice in the face and upper trunk	9	3.7
		TSB > PT limit of NICE guideline	10	4.1
		Others	2	0.8
2	Do you measure a predischarge TSB or TcB in all newborns?	Yes	17	6.9
		Yes, in jaundiced infants	115	46.3
		No	116	46.8
3	Do you perform a risk assessment by using the Bhutani nomogram before discharge?	Yes	102	41
		No	147	59
4	What is your preferred method of early recognition of neonatal jaundice?	Visual estimation using Kramer score	190	76
		TcB	7	2.8
		TSB	51	20.4
		Others	2	0.8
5	What will you do when a newborn with neonatal jaundice is referred to you?	Start phototherapy	37	14.8
		Stop breastfeeding	1	0.4
		Increase formula feeding	6	2.4
		Give parenteral fluid	2	0.8
		Observation and cancel discharge	12	4.8
		Plot TSB in a nomogram with PT thresholds	61	24.4
		Order TcB/TSB, blood group	131	52.4
6	What do you usually do in a formal assessment of a newborn with neonatal hyperbilirubinemia?	TSB only	8	3.2
		TSB and direct bilirubin (DB)	52	21
		TSB, DB, blood group (BG) of the mother and newborn	117	47.2
		TSB, DB, BG, G6PD, direct anti-globulin test (DAT)	26	10.5
		TSB, DB, BG, DAT, peripheral blood smear, reticulocyte, albumin	39	15.7
		TcB before PT	5	2
		TcB 24 h after PT stop	1	0.4
7	When will you start PT in a full-term or nearly full-term newborn (>35 weeks' GA)?	TSB >10 mg/dL for all hours of age	22	8.9
		Categorize low, moderate risk or high risk and plot TSB on an AAP guideline nomogram	187	75.7
		Clinical assessment using Kramer score more than Grade II-III	15	6.1
		Plot TSB according to the infant's age in hours and GA with NICE guideline	23	9.3
		Others	0	0
8	When will you start PT in preterm infants (<35 weeks' GA) ?	TSB >10 mg/dL in all hours of age	26	10.6%
		Categorize low, moderate, or high risk and plot TSB on an AAP guideline nomogram	162	65.9
		Clinical assessment using Kramer score more than Grade II-III	7	2.8
		TSB >0,5–0,7% of birth weight in healthy preterm (Fanaroff and Martin's) [4]	16	6.5
		TSB >0,4–0,6% of birth weight in sick preterm (Fanaroff and Martin's) [4]	11	4.5
		Plot TSB according to the infant's age in hours and GA with NICE guideline	24	9.8
		Others	0	0
9	What kind of PT lamp do you usually use in your hospital?	White lamp	17	6.9
		Fluorescent blue lamp	191	77.6
		LED	26	10.6
		Halogen	7	2.8
		Halogen fiber optic	2	0.8
		Gas discharge tube	0	0
		Others	3	1.2
10	Do you regularly measure the intensity of the PT lamps in your hospital?	Yes	26	10.6
		No	220	89.4
11	What do you usually do to increase the intensity of the PT?	Use double or even triple PT devices	196	79.4
		Covering the incubator/crib with curtains	30	12.1
		Covering the incubator/crib with aluminum foil	11	4.5
		Change the lamp	4	1.6
		Change the distance closer to the infants	6	2.4
		Others	0	0
12	What additional therapy do you give for neonatal hyperbilirubinemia?	Extra fluid intravenous	62	25.4
		Extra feeding	110	45.1
		Stop breastfeeding	8	3.3
		Albumin infusion	10	4.1
		Phenobarbitone	21	8.6
		Ursodeoxycholic acid (UDCA)	23	9.4
		Intravenous immunoglobulin (IVIG)	7	2.9
		Others	3	1.2
13	When do you stop PT?	TSB <10 mg/dL	113	46.5
		TSB >2 mg/dL below PT threshold	88	36.2
		Clinical assessment Kramer score of 1 or less	26	10.7
		If cholestasis to avoid bronze baby	9	3.7
		Others	7	2.9
14	What is your definition of hyperbilirubinemia requiring exchange transfusion for a full-term and nearly full-term infant (>35 weeks' GA)?	TSB >20 mg/dL in all hour's age	67	27
		Categorize low, moderate, or high risk and plot TSB on an AAP guideline nomogram	144	58.1
		Clinical assessment using Kramer score higher than Grade II-III	7	2.8
		Even low TSB with signs of hemolysis and positive DAT plus signs of bilirubin encephalopathy	14	5.6
		Plot TSB according to the infant's age in hours and GA with NICE guideline	16	6.5
		Others	0	0
15	What is your definition of hyperbilirubinemia requiring exchange transfusion in preterms (<35 weeks' GA)?	TSB >20 mg/dL in all hours of age	62	25.2
		Categorize low, moderate, or high risk and plot TSB on an AAP guideline nomogram	135	54.9
		Clinical assessment using Kramer score higher than Grade II-III	5	2
		Even low TSB but with signs of hemolysis and positive DAT plus signs of bilirubin encephalopathy	12	4.9
		TSB >1% of birth weight	3	1.2
		Plot TSB according to the infant's age in hours and GA with NICE guideline	29	11.8
		Others	0	0
16	Have you ever done an exchange transfusion?	Yes, please answer Questions 17, 18,19	99	40.1
		No, please skip Questions 17, 18, 19	148	59.9
17	What is your preferred route for an exchange transfusion?	Umbilical vein and umbilical artery	42	27.6
		Umbilical vein and peripheral artery	24	15.8
		Peripheral vein and peripheral Artery	33	21.7
		Peripheral vein only	38	25
		Umbilical vein - pull-push method	15	9.9
18	What kind of blood do you usually use for an exchange transfusion?	PRC, O- for Rh incompatible + FFP	7	4.7
		PRC, O Rh matching, low titer AB, for ABO incompatible + FFP	39	26.4
		Whole Blood, O- for Rh incompatible	21	14.2
		Whole Blood, O Rh matching, low titer AB	74	50
		PRC	7	4.7
19	What volume do you usually use when performing an exchange transfusion	Partial volume	11	7.4
		Single volume (80–90 mL/kg)	96	64.4
		Double volume (2 × 80–90 mL/kg)	38	25.5
		Others	4	2.7
20	What other treatments for hyperbilirubinemia do you use in your practice?	Sunlight therapy	114	47.1
		Probiotics	9	3.7
		D-penicillamine	4	1.7
		Clofibrate	1	0.4
		Sn-mesoporphyrin	1	0.4
		None	105	43.4
		Others	8	3.3
21	What do you usually do to monitor an infant with jaundice?	TSB every 6 h	18	7.3
		TSB every 12 h	25	10.2
		TSB every 24 h	179	73.1
		Others	23	9.4
22	When do you discharge a newborn with hyperbilirubinemia?	Clinical assessment, and infant in healthy condition, Kramer score Grade I, II or less	69	28
		TSB <5 mg/dL	18	7.3
		TSB <10 mg/dL	46	18.7
		Low Risk Category in Bhutani nomogram	62	25.2
		TSB below PT threshold	51	20.7
23	When do you schedule the neurodevelopmental follow-up of an infant following severe hyperbilirubinemia?	1 month of age	91	37.3
		3 month of age	106	43.4
		6 month of age	47	19.3
24	Do you have access to perform brainstemevoked response audiometry (BERA) for a patient with severe hyperbilirubinemia?	Yes	145	59.2
		No	100	40.8
25	Do you have access to perform growth developmental screening for a patient with severe hyperbilirubinemia?	Yes	182	74.6
		No	62	25.4

Abbreviations: AAP – American Academics of Paediatrics, TSB - total serum bilirubin, PT-phototherapy, TcB - transcutaneous bilirubin, DB - direct bilirubin, BG - blood group, DAT-direct antiglobulin test, G6PD – Glucose-6 Phosphate Dehydrogenase, LED – Light-Emitting Diode, NICE – The National Institute for Health and Care Excellence, GA – gestational age, PRC - packed red cells, FFP - fresh frozen plasma, AB - antibodies.

The Indonesian Pediatrics Society and the Indonesian Working Group on Neonatology both recommended using the AAP guideline on the prevention, diagnosis and treatment of hyperbilirubinemia in full-term and nearly full-term infants [5]. The entire AAP guideline was translated literally from English to Bahasa Indonesia and published in the guideline manual for medical pediatric services and the guideline manual for neonatology [6]. For hyperbilirubinemia management in preterm infants the guideline manual for neonatology included recommendations for Phototherapy (PT) and exchange transfusion (ET) limits based on the Manual of Neonatal Care by Cloherty et al [7].

Our questionnaire consisted of questions on the recognition and definition of hyperbilirubinemia, on the indications for PT and/or other therapies, and on the use of guidelines for starting and stopping PT. We also asked questions about the residents’ knowledge of and experience with ET. Finally, we inquired about their follow-up policy regarding infants with hyperbilirubinemia.

## 3 Results

We received 250 completed questionnaires out of the 368 we had handed out (68%), with a mean of 35 respondents from each hospital, ranging from 14 to 113 per hospital. In Table 1 we present the number and percentages of the responses to all questions. The definitions of hyperbilirubinemia varied largely: 25.6% of the respondents used a fixed TSB threshold, 19% used a fixed TSB threshold on the first or second day, and 45.9% used the postnatal age in hours and the TSB threshold of the Bhutani nomogram [8]. A pre-discharge TSB or TcB measurement was done by 46.3% of the respondents in jaundiced infants, but only 6.9% reported to do so for all infants. Seventy-six percent of the respondents used visual assessment, i.e. the Kramer score [9] to recognize jaundice, while 20.4% measured a TSB. A minority (2.8%) used transcutaneous bilirubin measurements [Table 2] [10]. On admission of a jaundiced newborn, 14.8% of the respondents would start PT before obtaining a TSB. Almost 77% of the respondents correctly answered that PT is indicated in full-term or nearly full-term infants by plotting the TSB on an AAP nomogram, 9.3% used the nomogram provided by the NICE guideline [11] and 14% used either a fixed TSB concentration or the Kramer score for this matter. Regarding the question on the indication to start PT in preterm infants, 66% of the respondents indicated that they plotted the TSB value on the AAP nomogram (i.e. on the nomogram developed for infants of more than 35 weeks’ gestation) and 10% on the gestational age-dependent nomogram of the NICE guideline and 24% used either a fixed TSB concentration (predefined or based on birth weight) or the Kramer score to start PT [Table 2].

**Table 2:** Diagnostic methods of hyperbilirubinemia as reported by residents in pediatrics in five Indonesian academic teaching hospitals.

Questions and Answers		Hospitals				
		A	B	C	D	E
		n(%)	n(%)	n(%)	n(%)	n(%)
What is your preferred method for early recognition of neonatal jaundice?	Visual estimation using the Kramer score	11 (31.4)	20 (83.3)	9 (64.3)	46 (71.9)	104 (92.0%)
	TcB	7 (20)	0 (0)	0 (0)	0 (0)	0 (0%)
	TSB	16 (45.7)	4 (16.7)	5 (35.7)	18 (28.1)	8 (7.1%)
	Others	1 (2.9)	0 (0)	0 (0)	0 (0)	1 (0.9%)
Do you measure TSB and perform a risk assessment by using the Bhutani nomogram before discharge?	Yes	9 (25.7)	16 (66.7)	7 (50)	30 (47.6)	40 (35.4%)
	No	26 (74.3)	8 (33.3)	7 (50)	33 (52.4)	73 (64.6%)
What is your definition of hyperbilirubinemia?	TSB> 5 mg/dL in 24 h, 10 mg/dL in 48 h	8 (22.9)	6 (25)	6 (42.9)	7 (11.3)	22 (19.8)
	TSB 10 mg/dL for full-term infants, and TSB >5 mg/dL for preterms	14 (40)	3 (12.5)	1 (7.1)	3 (4.8)	42 (37.8)
	TSB > P_95_ according to hours of age (Bhutani nomogram)	5 (14.3)	8 (33.3)	4 (28.6)	27 (43.5)	34 (30.6)
	TSB-P_40_-P_75_ according to hours of age (Bhutani nomogram)	1 (2.9)	0 (0)	1 (7.1)	7 (11.3)	6 (5.4)
	TSB > P_5_ according to hours of age (Bhutani nomogram)	0 (0)	6 (25)	1 (7.1)	8 (12.9)	5 (4.4)
	Clinical assessment with jaundice in the face and upper trunk	0 (0)	1 (4.2)	1 (7.1)	7 (11.3)	0 (0)
	TSB > PT limit of the NICE guideline	7 (20)	0 (0)	0 (0)	2 (3,2)	1 (0,9)
	Other	0 (0)	0 (0)	0 (0)	1 (1,6)	1 (0,9)

Hospitals A to E in random order. Abbreviations: TcB - transcutaneous bilirubin, TSB - total serum bilirubin, PT - phototherapy.

PT was usually provided by white or fluorescent blue lamps. Hospital A, C, D and E have fluorescent and LED devices. Hospital B has LED PT devices. Only 10.6% of the respondents reported using LED-based PT devices. Intensity measurements were rarely performed. Regarding additional therapy besides PT, 70.5 % of the respondents reported administering extra fluids, either orally or intravenously, and 25% indicated using additional medications, including albumin, phenobarbitone, ursodeoxycholic acid or intravenous immunoglobulins [Table 3].

**Table 3:** Management of hyperbilirubinemia as reported by resident in pediatrics in five Indonesian academic teaching hospitals.

Questions and answers		Hospitals				
		A	B	C	D	E
		n(%)	n(%)	n(%)	n(%)	n(%)
What will you do when a newborn with neonatal jaundice is referred to you?	Start PT	4 (11)	0 (0)	2 (14)	3 (5)	28 (25)
	Stop breastfeeding	1 (3)	0 (0)	0 (0)	0 (0)	0 (0)
	Increase formula feeding	6 (17)	0 (0)	0 (0)	0 (0)	0 (0)
	Give parenteral fluids	2 (6)	0 (0)	0 (0)	0 (0)	0 (0)
	Observation and cancel discharge	0 (0)	0 (0)	1 (7)	0 (0)	11 (10)
	Plot TSB to nomogram with PT thresholds	13 (37)	9 (38)	4 (29)	10 (15)	25 (22)
	Order TcB/TSB, blood group	9 (26)	15 (62)	7 (50)	51 (80)	49 (43)
When will you start PT in a full-term or nearly full-term newborn (>35 week GA) ?	TSB >10 mg/dL in all hours of age	5 (14)	1 (4)	1 (7)	0 (0)	15 (14)
	Categorize low, moderate or high risk and plot TSB on an AAP guideline nomogram	22 (63)	23 (96)	13 (93)	61 (95)	68 (61)
	Clinical assessment using Kramer score more than Grade II-III	0 (0)	0 (0)	0 (0)	0 (0)	15 (14)
	Plot TSB according to the age in hours and GA with NICE guideline	8 (23)	0 (0)	0 (0)	3 (5)	12 (11)
When will you start PT in a preterm newborn (<35 weeks' GA) ?	TSB >10 mg/dL in all hours age	3 (9)	1 (4)	6 (43)	1 (2)	15 (14)
	Categorize low, moderate or high risk and plot TSB on an AAP guideline nomogram	19 (54)	18 (75)	6 (43)	52 (82)	67 (61)
	Clinical assessment using Kramer Score more than grade II-III	0 (0)	1 (4)	0 (0)	0 (0)	6 (6)
	TSB is 0,5–0,7% of birth weight in healthy preterm (Fanaroff Martin's guideline) [4]	5 (14)	1 (4)	0 (0)	3 (4)	7 (5)
	TSB is 0,4–0,6% of birth weight in sick preterm (Fanaroff Martin's guideline) [4]	5 (14)	0 (0)	1 (7)	1 (2)	4 (4)
	Plot TSB according to age in hours and GA with NICE guideline	3 (9)	3 (13)	1 (7)	6 (10)	11 (10)
What kind of PT lamp do you usually use in your hospital?	White lamp	3 (8.6)	0 (0)	0 (0)	1 (1.6)	13(11.8)
	Fluorescent blue lamp	18 (51.4)	21 (87.5)	12 (85.7)	56 (88.9)	84 (76.4)
	LED	9 (25.7)	3 (12.5)	1 (7.1)	5 (7.9)	8 (7.3)
	Halogen	3 (8.6)	0 (0)	1 (7.1)	1 (1.6)	2 (1.8)
	Halogen fiber optic	2 (5.7)	0 (0)	0 (0)	0 (0)	0 (0)
	Gas discharge tube	0 (0)	0 (0)	0 (0)	0 (0)	0 (0)
	Others	0 (0)	0 (0)	0 (0)	0 (0)	3 (2.7)
Do you regularly measure the intensity of the PT lamps in your hospital?	Yes	7 (20)	0 (0)	5 (35.7)	7 (11.1)	7 (6.4)
	No	28 (80)	24 (100)	9 (64.3)	56 (88.9)	103 (93.6)
What do usually do to increase the intensity of the PT?	Use double or even triple PT devices	21 (0)	24 (100)	10 (71.4)	54 (84.4)	87 (79.1)
	Covering the incubator/crib with curtains	10 (28.6)	0 (0)	2 (14.3)	4 (6.3)	14 (12.7)
	Covering the incubator/crib with aluminum foil	2 (5.7)	0 (0)	1 (7.1)	3 (4.7)	5 (4.5)
	Change the lamp	2 (5.7)	0 (0)	1 (7.1)	0 (0)	1 (0.9)
	Change the distance closer to the infants	0 (0)	0 (0)	0 (0)	3 4.7)	3 (2.7)
	Others	0 (0)	0 (0)	0 (0)	0 (0)	0 (0)
What additional therapy do you give for neonatal hyperbilirubinemia?	Extra fluid intravenous	6 (17.1)	1 (4.2)	3 (21.4)	38 (62.3)	14 (12.7)
	Extra feeding	20 (57.1)	15 (62.5)	11 (78.6)	18 (29.5)	46 (41.8)
	Stop breastfeeding	5 (14.3)	0 (0)	0 (0)	0 (0)	3 (2.7)
	Albumin infusion	1 (2.9)	0 (0)	0 (0)	1 (1.6)	8 (7.3)
	Phenobarbitone	0 (0)	1 (4.2)	0 (0)	1 (1.6)	19 (17.3)
	Ursodeoxycholic acid (UDCA)	1 (2.9)	6 (25)	0 (0)	1 (1.6)	15 (13.6)
	Intravenous immunoglobuline (IVIG)	2 (5.7)	1 (4.2)	0 (0)	0 (0)	4 (3.6)
	Others	0 (0)	0 (0)	0 (0)	2 (3.3)	1 (0.9)
When do you stop PT?	TSB <10 mg/dL	20 (57.1)	17 (70.8)	4 (30.8)	13 (21.3)	59 (53.6)
	TSB >2 mg/dL below threshold for PT	9 (25.7)	4 (16.7)	9 (69.2)	36 (59)	30 (27.3)
	Clinical assessment Kramer score of 1 or less	2 (5.7)	3 (12.5)	0 (0)	5 (8.2)	16 (14.5)
	If cholestasis, to avoid bronze baby	3 (8.6)	0 (0)	0 (0)	4 (6.6)	2 (1.8)
	Others	1 (2.9)	0 (0)	0 (0)	3 (4.9)	3 (2.7)

Hospitals A to E in random order. Abbreviations: TSB - total serum bilirubin, TcB - transcutaneous bilirubin, PT-phototherapy.

The majority of the respondents answered the question on the indication for ET in full-term infants correctly: 63.7% used the AAP guideline and/or signs of acute bilirubin encephalopathy and 6.5% used the NICE guideline, whereas 27% used a fixed TSB cut-off value of 20 mg/dL (340 μmol/L) and 2.8% based their decisions to perform an ET on visual assessment only. The question on the threshold for an ET in preterm infants was answered correctly by a minority of the respondents: 11.8% used the NICE guideline, whereas 25.2% used a fixed cut-off value of 20 mg/dL, 1.2% used a TSB value >1% of birth weight (1.2%) and 2% used the Kramer score. The majority (54.9%) referred to a nomogram of the AAP guideline that is recommended for near full-term and full-term infants.

More than half of the respondents, 59.9%, had never performed an ET and only 26.4% correctly answered the questions about the composition of the fluid components for an ET and 25.5% answered the question on the recommended ET volume correctly [Table 3].

Sunlight PT was mentioned by 47.1% of the respondents as an alternate treatment for neonatal hyperbilirubinemia and 6.2% reported the use of probiotics, D-penicillamine, clofibrate or Sn-mesoporphyrin. There were remarkable differences in the responses to questions regarding the preferred diagnostic methods and therapeutic management between the respondents from different teaching hospitals [Table 2 and Table 3].

## 4 Discussion

Our study indicates that the knowledge of pediatric residents in teaching hospitals in Indonesia on the management of hyperbilirubinemia in full-term and preterm newborn infants is limited and their use of management guidelines is variable.

A remarkable finding was that a significant percentage of the residents did not measure a TSB before starting PT, but instead used the clinical evaluation by the Kramer score to diagnose hyperbilirubinemia. The time between taking blood for a TSB measurement and obtaining the result was approximately 1 h for hospital B and 3 h for hospitals C and E. They did not have bedside TcB that shows results within few minutes. These problems might contribute their decision to start phototherapy regardless the TSB value. This approach is often used by the residents because, besides the AAP and NICE guidelines, the recommendations originating from the WHO are in use in Indonesia [12]. The Indonesian Ministry of Health issued a guideline based on the WHO publication named Pocketbook of Hospital Care for Children, which is a compilation of guidelines for the management of common childhood illnesses as the first referral level in low-resource countries with basic laboratory facilities and inexpensive medicines [13]. The Indonesian Pediatric Society also issued *Panduan Pelayanan Medik* that mostly is translated from the AAP guideline which is used by many residents and pediatricians. The WHO recommendations are often used in primary care by midwifes and general practitioners and recommend starting PT on the basis of visual assessment (a high Kramer score) when a TSB measurement is not accessible. While this approach is understandable in remote areas with limited resources, it is not acceptable in academic centers where the infra sructure to obtain TSB is available. Still, in well-equipped teaching hospitals some residents apply this WHO recommendation, which was intended specifically for basic circumstances. It appears that residents use a random selection of recommendations stemming from different guidelines, rather than using the complete set of recommendations from one guideline, be it AAP or NICE. These guidelines stress that a clinical evaluation by the Kramer score cannot differentiate between hyperbilirubinemia that needs to be treated or not [5,11]. Transcutaneous bilirubin measurements could serve as a good screening method, because it is reliable and shows good correlations with TSB values [10]. Yet, only two of the five teaching hospitals in our study used this method. Only Hospital C and D have TcB as a non-invasive tool for estimating hyperbilirubinemia. But the use is still limited for research purposes and not as a routine. Therefore, residents are often not aware of the possibilities and limitations of transcutaneous bilirubin screening.

Most respondents reported that the hospitals in Indonesia still use conventional PT devices equipped with fluorescent tubes (FT) as is the case in many other low and middle-income countries around the world [14]. But these have several disadvantages compared to the newer LED PT devices, such as more heat production, lower sustainability and less reliable irradiance performance. The latter is of great importance for PT to be effective. Nevertheless, by far the majority of respondents reported that irradiance was seldom measured and that the intensity of PT was pragmatically increased by adding an extra device or two (double or triple PT). Nowadays, LED devices are not more expensive than FT devices, thus the use of LED devices will probably increase in the near future. The use of radiometers to control the output of the PT devices is highly recommended. Measurement of the irradiance of PT devices will not only improve the quality of PT, but will also enhance the understanding of the principles of PT.

Another remarkable finding was the significant percentage of respondents who reported prescribing adjuvant medicines, such as ursodeoxycholic acid and phenobarbital, which are not recommended by the AAP and NICE for neonatal jaundice nor by the WHO guideline. We think that either residents copy prescription habits of supervising pediatricians who have apparently developed their personal treatment strategies that do not comply with international, evidence-based, guidelines. Or, alternatively, the pediatricians do not succeed in explaining to their residents what the specific indications are to prescribe these off-label drugs, or the residents fail to understand.

Another alternative treatment that respondents reported using was sunlight. Direct sunlight is often used in low-income countries if conventional PT is not available. Although filtered sunlight may be an option, direct sunlight is not safe due to the high levels of infrared and UV radiation that could lead to overheating, dehydration and sunburn. Therefore, sunlight PT is not recommended when electric powered PT is available [5,11,15]. Again this may reflect a discrepancy between the development of the level of health care in Indonesia and alternative methods that could be used in circumstances with fewer medical resources [15].

Guidelines may not be adhered to for a number of reasons [16]: lack of awareness, lack of familiarity, lack of agreement, lack of self-efficacy, lack of outcome expectancy, lack of motivation to change current practice, and external barriers. Most of these reasons are probably applicable to the pediatric residents in Indonesia. For instance, our specific finding that 66% of the respondents indicated using the AAP guideline when they considered starting PT in preterm infants, while the AAP guideline does not contain such a nomogram for preterm infants born before 35 weeks’ gestation, could be an example of lack of familiarity with that guideline. Another frequently applied method was using a fixed bilirubin level to start PT. This may reflect a lack of motivation to gather the additional information, such as hour of birth, exact gestational age (GA) and risk factors, needed to follow the recommended decision criteria whether PT is indicated or not. The frequent use of additional, non-recommended medication could be a reflection of lack of self-confidence or lack of outcome expectancy of PT.

A lack of proper training may also account for how the residents’ performance. Proper training is a responsibility of both the tresident and the trainer. The trainers have to supervise the residents to follow the applicable guideline. Apparently they have failed in this regard. The guideline already exists, even in Indonesian language, and available widespread.Or it is possible that the trainers themselves do not comply with the current guideline. Since the residents have a master companion relationship with the trainers, they will automatically follow the behaviour of the trainers. In this case, the trainers should also reflect on themselves and realize that they seemed to have failed in their training of management of hyperbilirubinemia. Proper training is necessary to lower the incidence of severe hyperbilirubinemia. When the knowledge of the pediatricians about the management of hyperbilirubinemia and the infrastructure for diagnosis, therapy and referral are optimal, then the occurrence of severe hyperbilirubinemia and its sequelae will be limited. For this reason, we need to warn our colleagues to duly adjust and improve the educational program. Trainers must carry out self reflections about the methods and techniques in teaching and practice with regard to the management of hyperbilirubinemia.

In addition, consensus is needed among trainers in academic teaching hospital to obtain a uniform method of training residents in the management of hyperbilirubinemia. Furthermore, additional research is needed to explore these barriers to be able to improve residents’ adherence to guidelines and to improve the quality of care for newborns with hyperbilirubinemia. The questionnaire will be used following a quality improvement cycle, according to the PDCA cycle (Plan-Do-Check-Act) after introduction of web-based application together with the implemantation of a new Indonesian Guideline for the diagnosis and management of hyperbilirubinemia (Suparma, submitted).

We think that some of our findings are probably not confined to Indonesian pediatric residents alone, as comparable results were found for pediatricians in high income countries. A study by Petrova et al, conducted in the USA, reported that the majority of pediatricians did not consider neonatal jaundice after discharge and GAs of 37–38 weeks as being significant risk factors for developing severe hyperbilirubinemia [17]. Almost 60% of these pediatricians, however initiated PT at TSB levels lower than recommended by the AAP in newborns more than 72 h of age, thus confirming that pediatricians do not always adhere to hyperbilirubinemia guidelines. The Canadian studies by Darling and Sgro et al, however, showed that it is possible to overcome the barriers to successfully implementing a national hyperbilirubinemia guideline and that this indeed resulted in a lower incidence of severe hyperbilirubinemia [2,3].

Our study has limitations. First, we have no record of the number of residents in each hospital, so we do not know the percentage of residents participating in this survey. We think however that it is not likely that residents who are well trained in the management of hyperbilirubinemia would not be inclined to participate in this survey. Secondly, we did not include questions regarding the type of training or supervision by supervisors. More study is needed regarding the role of supervisors in obtaining the proper knowledge by the residents.

## 5 Conclusion

In conclusion, we found that the level of knowledge of residents in pediatrics in five teaching hospitals in Indonesia regarding the management of hyperbilirubinemia is limited and the use of management guidelines is variable and selective. This lack of kowledge is a common responsibility of the residents and the trainers. Trainers either might have insufficient knowledge of the state of art how to diagnose and treat hyperbilirubinemia, or they do not sufficiently teach and supervise the residents. General knowledge upgrade for the trainers and residents is needed regarding the diagnostic process and treatment of hyperbilirubinemia, as well as exploring what the barriers are to following the guidelines. One national guideline on the management of hyperbilirubinemia that is adapted to the Indonesian health care system integrating wih web-based application could improve the adherence to the guideline and will help towards reducing the incidence of severe hyperbilirubinemia.

## Declarations

### Author contribution statement

Mahendra Tri Arif Sampurna: Conceived and designed the experiments; Performed the experiments; Analyzed and interpreted the data; Contributed reagents, materials, analysis tools or data; Wrote the paper.

Rinawati Rohsiswatmo, Aris Primadi, Setya Wandita and Eko Sulistijono: Performed the experiments; Contributed reagents, materials, analysis tools or data.

Arend F Bos, Pieter JJ Sauer, Christian V Hulzebos and Peter H Dijk: Analyzed and interpreted the data; Contributed reagents, materials, analysis tools or data.

### Funding statement

This research did not receive any specific grant from funding agencies in the public, commercial, or not-for-profit sectors.

### Data availability statement

Data will be made available on request.

### Declaration of interests statement

The authors declare no conflict of interest.

### Additional information

No additional information is available for this paper.
